# S100A7 promotes lung adenocarcinoma to squamous carcinoma transdifferentiation, and its expression is differentially regulated by the Hippo-YAP pathway in lung cancer cells

**DOI:** 10.18632/oncotarget.15063

**Published:** 2017-02-03

**Authors:** Rui Wang, Yunguang Li, Enze Hu, Fei Kong, Junhao Wang, Jin Liu, Qirui Shao, Ying Hao, Dacheng He, Xueyuan Xiao

**Affiliations:** ^1^ Key Laboratory of Cell Proliferation and Regulation Biology, Ministry of Education, College of Life Science, Beijing Normal University, Beijing, China; ^2^ The Department of Basic Theory, College of Sports, Northwest Normal University, Lanzhou, China

**Keywords:** S100A7, lung cancer cells, ADC to SCC transdifferentiation, YAP, the Hippo pathway

## Abstract

Our previous study revealed that S100A7 was selectively expressed in lung squamous cell carcinoma tissues but not in adenocarcinoma. Thus far, the functions of S100A7 in lung cancer have remained largely unknown. Here, we reveal that S100A7 overexpression facilitates the transdifferentiation from adenocarcinoma (ADC) to squamous carcinoma (SCC) in several lung cancer cells, which is confirmed by an increase in DNp63 expression and a decrease in thyroid transcription factor 1 (TTF1) and aspartic proteinase napsin (napsin A) expression. Further study finds that activation of the Hippo pathway induces S100A7 expression and further confirms that nuclear YAP acts as a repressor of S100A7 in H292 cells. Subsequently, we verify that TEAD1 is required for YAP transcriptional repression of S100A7. More importantly, we determine that S100A7 overexpression partially rescues lung ADC to SCC transdifferentiation inhibited by YAP overexpression in all tested cells, suggesting that S100A7 and YAP have the opposite effects on lung ADC to SCC conversion. Taken together, our study demonstrates for the first time that S100A7 not only functions as a facilitator of adenous-squamous carcinoma phenotypic transition in lung cancer cells but also that its expression is differentially regulated by the Hippo-YAP pathway.

## INTRODUCTION

Human lung cancer is the leading cause of worldwide cancer-related mortality, and the incidence is progressively increasing in China. Non-small cell lung cancer (NSCLC) and small cell lung cancer (SCLC) are two major groups of lung cancers, and the former further comprises adenocarcinoma (ADC), squamous cell carcinoma (SCC) and large cell carcinoma [1, 2, 3]. However, lung ADC and SCC are considered to arise from different epithelial cells in the lung. Accordingly, lung ADC mainly expresses TTF1 and napsin A, whereas lung SCC expresses DNp63 and cytokeratin 5/14 [4, 5]. Interestingly, adenosquamous carcinoma (mixed ADC and SCC) has been frequently observed in single lesions of human lung tumors, suggesting a monoclonality and a potential lineage transition between these two subtypes [6]. Although the precise mechanism of lineage correlation or dynamic transition between human lung ADC and SCC remains largely unknown, recent reports have provided direct evidence to support ADC to SCC transdifferentiation in Lkb1-deficient mouse models of human lung cancer [6, 7].

S100A7 belongs to the S100 multigenic family of calcium-modulated proteins of the EF-hand type and was originally identified in psoriatic keratinocytes [8, 9, 10, 11, 12]. Subsequent studies have shown that upregulation of S100A7 is detected in nearly all types of SCC tissues as well as adenocarcinomas of the breast [13, 14, 15, 16, 17]. Our previous study showed that S100A7 was selectively expressed in lung SCC and large cell carcinoma tissues but not in ADC specimens [18, 19]. We further confirmed that S100A7 could be dramatically induced in several SCC cells and xenografts and acted as a dual regulator in promoting proliferation and suppressing squamous differentiation [20, 21]. However, little is known about how and why S100A7 induction occurs in lung cancer cells. Thus, understanding the mechanisms of S100A7 induction in lung cancer might have important implications for S100A7-targeted therapies.

The Hippo pathway was initially identified in *Drosophila* and is an important regulator of organ size through its tight control of cell growth and proliferation [22]. At the core of this pathway in mammals is a kinase cascade consisting of MST1/2 and LATS1/2. When the Hippo pathway is activated, MST1/2 phosphorylates the hydrophobic motif of LATS1/2 (LATS-HM) and activates LATS1/2 [23], which in turn directly phosphorylate YAP (Yes-associated protein) at serine 127 (YAP-S127) [24, 25, 26, 27]. The phosphorylation of YAP-S127 is inactivated through its cytoplasmic retention. Conversely, inactivation of the Hippo pathway leads to YAP nuclear translocation and downstream target gene expression through the binding of YAP to TEADs (the TEAD/TEF family transcription factors), the primary transcription factor partners of YAP, resulting in cell survival and proliferation [26, 27, 28, 29]. Recently, the Hippo pathway has also been found to regulate cell fate determination. For example, YAP inhibited squamous transdifferentiation of Lkb1-deficient lung adenocarcinoma through ZEB2-dependent DNp63 repression [7]. Moreover, our recent findings showed that YAP repressed S100A7 induction in A431 cells through activation of the Hippo pathway [29]. Therefore, it would be interesting to investigate the relationships and functions of YAP and S100A7 in other cancers, such as lung cancer.

Here, we verify that S100A7 acts as a facilitator of adenous-squamous phenotypic transition in lung cancer cells. We further demonstrate that S100A7 is not only induced by activation of the Hippo pathway but also that its overexpression partially rescues squamous differentiation inhibited by YAP overexpression in several lung cancer cells. Collectively, our findings may provide new insight into our understanding of the molecular basis of lung ADC to SCC transdifferentiation.

## RESULTS

### S100A7 promotes adenocarcinoma to squamous carcinoma transdifferentiation in lung cancer cells

**Figure 1:**
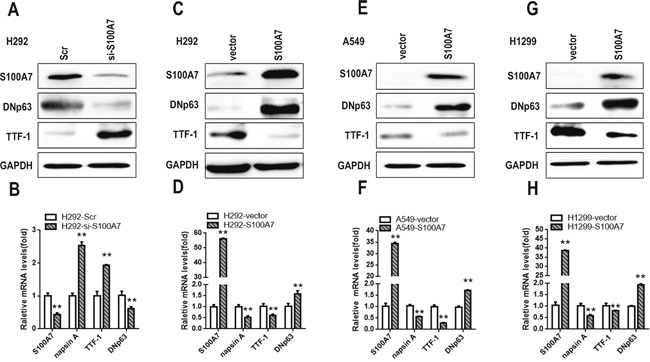
S100A7 promotes adenous to squamous transdifferentiation in lung cancer cells Depletion of S100A7 using siRNAs in H292 cells **A**. or overexpression of S100A7, DNp63 and TTF1 in H292 cells **B**., A549 cells **E**. and H1299 cells **G**. was examined by Western blotting. The expression of S100A7, DNp63, TTF1 and napsin A was detected by real time PCR **C, D, F**, and **H**., respectively. GAPDH was used to assess equal loading. **p < 0.01; t-test versus the experimental groups and the control groups.

Our previous study revealed that S100A7 was selectively expressed in lung SCC tissues but not in ADC tissues. Recent reports regarding lung ADC to SCC phenotypic transition in an Lkb1 (Liver kinase B1 or Serine-Threonine Kinase 11, STK 11) -deficent mouse model caught our attention [6]. To investigate whether S100A7 was involved in this transition process in lung cancer cells, three lung adenocarcinoma cell lines (H292, A549, and H1299 cells) were selected. Although the H292 cell line is a mucoepidermoid pulmonary carcinoma cell line that belongs to one subtype of adenocarcinoma, it expresses multiple markers of squamous differentiation according to the ATCC. Additionally, we found that H292 cells could express S100A7, but A549 and H1299 cells did not. Considering the expression levels of S100A7 in the different cell lines, we first depleted S100A7 in H292 cells (Figure 1A). Indeed, the SCC marker DNp63 was significantly downregulated, and the adenocarcinoma markers TTF1 and napsin A were markedly upregulated (Figure 1B), suggesting that silencing of S100A7 attenuated lung ADC to SCC transdifferentiation. Next, we found that overexpression of S100A7 inversely promoted this transition in the same cells (Figure 1C and 1D). Strikingly, introduction of S100A7 into A549 and H1299 cells also facilitated ADC to SCC conversion (Figure 1E, 1F, 1G and 1H). These results indicate that S100A7 has a promoting effect on ADC to SCC transdifferentiation in lung cancer cells.

### S100A7 is negatively regulated by YAP through activation of the Hippo pathway

**Figure 2:**
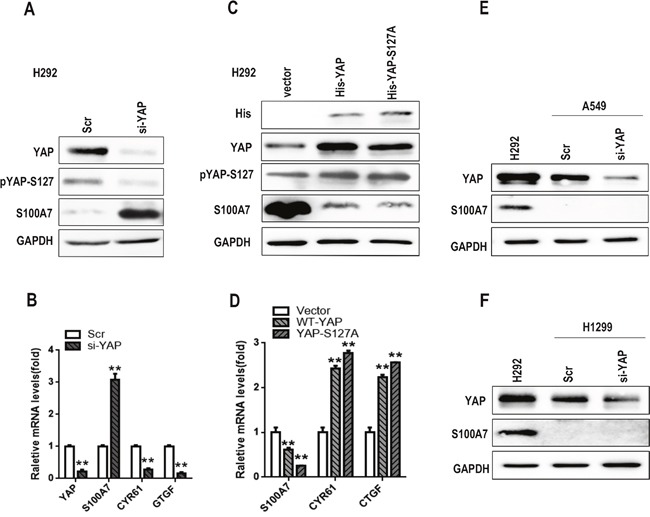
Knockdown of YAP promotes S100A7 expression in H292 cells To assess the depletion of YAP using siRNA in H292 cells, the expression of total YAP, p-YAP and S100A7 was examined by Western blotting **A**. The expression of YAP, S100A7, CYR61 and CTGF was determined by real-time PCR **B**. H292 cells were cultured in suspension for 48 h after overexpression of YAP-WT and YAP-S127A; the expression of YAP, pYAP-S127 and S100A7 was detected by Western blotting **C**. The expression of S100A7, CYR61 and CTGF was examined by real-time PCR **D**. Silencing of YAP with siRNA was also performed in A549 **E**. or H1299 **F**. cells, and the expression of YAP and S100A7 was determined by Western blotting; H292 cells served as a positive control. GAPDH was used to assess equal loading. **p < 0.01; t-test versus the experimental groups and the control groups.

**Figure 3:**
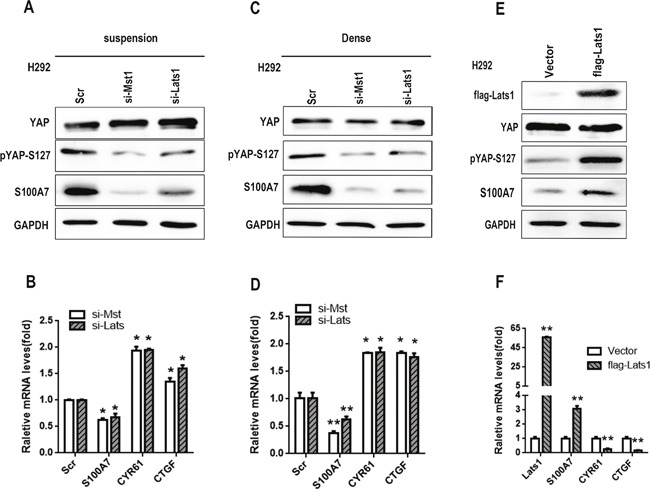
S100A7 is negatively regulated by YAP through activation of the Hippo pathway H292 cells were transfected with MST or Lats1 siRNAs, and the cells were then cultured in suspension **A**. or dense culture **C**. for 48 h. The expression of YAP, pYAP-S127 and S100A7 was detected by Western blotting. The expression of S100A7, CYR61 and CTGF was determined by real-time PCR **B, D**. Overexpression of LATS1 in H292 cells; an anti-Flag tag antibody was used to judge the transfection efficiency. The expression of YAP, pYAP-S127, S100A7, *CYR61* and *CTGF* was examined by Western blotting **E**. or real-time PCR **F**. GAPDH was used to assess equal loading. *p < 0.05, **p < 0.01; t-test versus the experimental groups and the control groups.

A recent study showed that overexpression of YAP inhibited ADC to SCC transdifferentiation of human lung cancer in an Lkb1-deficient mouse model, whereas knockdown of YAP facilitated squamous transdifferentiation [7]. Together, the above results and the inhibitory effect of YAP on S100A7 expression in A431 cells [30] gave us reason to speculate that YAP most likely functions as a repressor of S100A7 in H292 cells. To test this, we depleted YAP expression in H292 cells using specific siRNA. As expected, single depletion of YAP was sufficient to induce the expression of S100A7 mRNA and protein, and the efficiency of YAP knockdown was also confirmed by a decrease in CTGF (Connective tissue growth factor) and CYR61 (Cysteine-rich angiogenic inducer 61) expression (Figure 2A and 2B). Conversely, the constitutively active YAP mutant YAP-S127A and YAP-WT markedly inhibited S100A7 expression in the same cells, and the former had a greater effect on S100A7 repression than the latter (Figure 2C and 2D), suggesting that nuclear YAP is a repressor of S100A7 expression in H292 cells. However, S100A7 induction was not detected in A549 and H1299 cells with YAP knockdown (Figure 2E and 2F), suggesting that additional genetic or epigenetic changes were also required for S100A7 induction in these cells. We further found that knockdown of LATS1 and MST1 rescued the inhibitory effect of YAP on S100A7 expression in suspended or densely cultured H292 cells (Figure 3A, 3B, 3C and 3D), whereas overexpression of LATS1 led to an increase in S100A7 expression and YAP phosphorylation (Figure 3E and 3F). Together, our data unequivocally demonstrate that activation of the Hippo pathway represses S100A7 expression in H292 cells.

### F-actin disruption promotes S100A7 expression and YAP phosphorylation

**Figure 4:**
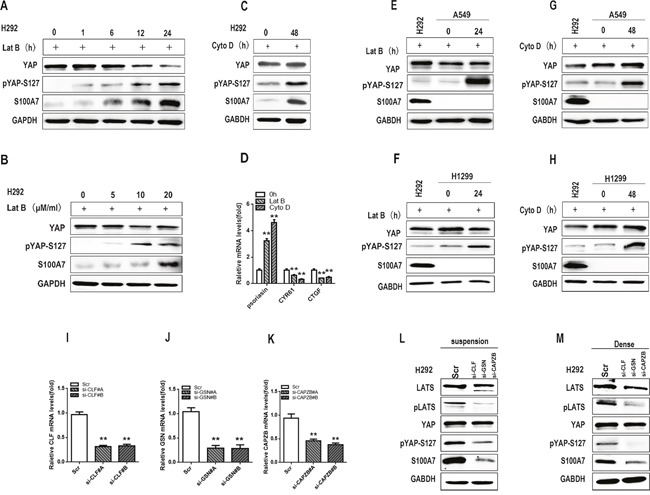
F-actin disruption promotes S100A7 expression and YAP phosphorylation H292 cells were treated with Lat B (20 μg/ml) for different lengths of time **A**. and at different doses **B**. H292 cells were also treated with Cyto D (0.05 μM) for 48 h **C**., and the expression of total YAP, p-YAP and S100A7 was examined by Western blotting. The expression of S100A7, CYR61 and CTGF was determined by real-time PCR **D**. A549 and H1299 cells were also treated with Lat B (20 μg/ml) for 24 h **E, F**. and Cyto D (0.05 μM) **G, H**. for 48 h. The expression of total YAP, p-YAP and S100A7 was examined by Western blotting, and H292 cells served as a positive control. The silencing efficiency of CLF, GSN and CAPZB was examined by real-time PCR **I, J, K**. H292 cells were transfected with CLF, GSN and CAPZB siRNAs, and the cells were then cultured in suspension **L**. or dense culture **M**. for 48 h; the expression of LATS, pLATS, YAP, pYAP-S127 and S100A7 was detected by Western blotting. GAPDH was used to assess equal loading. **p < 0.01; t-test versus the experimental groups and the control groups.

Cell suspension and dense culture also lead to activation of the Hippo pathway through actin cytoskeleton remodeling in several cell lines [31, 32]. To investigate whether F-actin cytoskeletal reorganization participated in S100A7 expression and YAP phosphorylation in H292 cells, two F-actin cytoskeleton-disrupting reagents, latrunculin B (Lat B) and cytochalasin D (Cyto D), were used. Lat B and Cyto D disrupt the F-actin cytoskeleton by preventing actin polymerization and by capping filament plus ends, respectively [33]. As expected, abrogation of F-actin polymerization by Lat B resulted in S100A7 induction and YAP phosphorylation in a time- and dose-dependent manner (Figure 4A and 4B), suggesting that F-actin integrity plays a critical role in the control of S100A7 expression and YAP activity in H292 cells. Similar results were also observed in the same cells after Cyto D treatment (Figure 4C). S100A7 expression and YAP inactivation were also confirmed by qPCR (Figure 4D). Unsurprisingly, neither Lat B nor Cyto D treatment resulted in S100A7 induction in A549 or H1299 cells (Figure 4E, 4F, 4G and 4H). To further confirm whether F-actin plays an important role in S100A7 induction, we knocked down Cofilin1 (CFL1), Gelsolin (GSN) and CAPZB together in attached H292 cells using their specific siRNAs (Figure 4I, 4J and 4K) and then cultured cells in suspension and dense for two days. As expected, S100A7 induction was significantly blocked and YAP-S127 was also markedly decreased in silenced cells compared with control cells (Figure 4L and 4M). Taken together, these data support the notion that reorganization of the actin cytoskeleton plays an important role in S100A7 induction through activation of the Hippo pathway.

### TEAD1 is indispensable for YAP-repressed S100A7 induction

**Figure 5:**
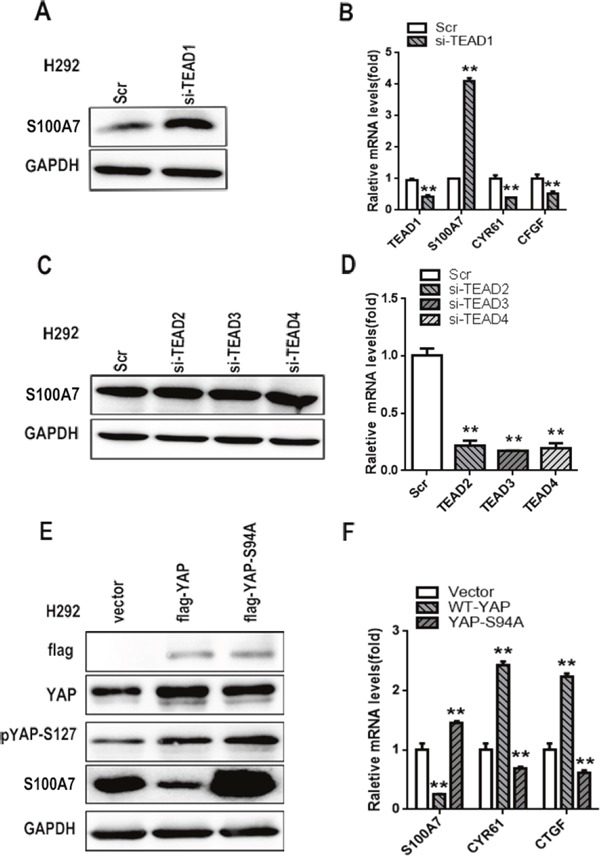
TEAD1 is indispensable for YAP-mediated S100A7 repression Knockdown of TEAD1 using siRNA in H292 cells **A**. The expression of TEAD1, S100A7, CYR61 and CTGF was determined by real-time PCR **B**. Knockdown of TEAD2, TEAD3, and TEAD4 using specific siRNAs in H292 cells. The expression of S100A7 was determined by Western blotting **C**. the silencing efficiency of TEAD2/3/4 was examined by real-time PCR **D**. H292 cells were transfected with YAP-WT and YAP-S94A plasmids. Then, the cells were cultured in suspension for 48 h, and the expression of YAP, pYAP-s127 and S100A7 was detected by Western blotting **E**. The expression of S100A7, CYR61 and CTGF was determined by real-time PCR **F**. GAPDH was used to assess equal loading. **p < 0.01; t-test versus the experimental groups and the control groups.

YAP does not have DNA-binding activity, and TEADs have emerged as one of the main partners of YAP binding to DNA to stimulate or repress YAP-dependent gene expression [34]. To investigate whether a TEAD was a YAP co-activator for S100A7 induction, we knocked down the expression of TEAD1/2/3/4 individually in H292 cells. Interestingly, transient depletion of TEAD1 alone was sufficient to induce S100A7 expression and inhibit CTGF and CYR61 expression (Figure 5A and 5B). Unexpectedly, knockdown of TEAD 2/3/4 did not facilitate S100A7 induction (Figure 5C and 5D). To further confirm this result, we overexpressed YAP-WT and YAP-S94A in H292 cells because YAP-S94A, unlike wild-type YAP and YAP-S127A, is defective in TEAD1 binding [35]. As expected, we found that S100A7 was marginally decreased by YAP-S94A overexpression compared with the control cells (Figure 5E and 5F), indicating that YAP inhibited S100A7 expression through interaction with TEAD1. Taken together, we conclude that TEAD1 functions as the transcriptional cofactor of YAP to repress S100A7 expression in H292 cells.

### S100A7 partially rescues lung ADC to SCC transdifferentiation inhibited by YAP overexpression

**Figure 6:**
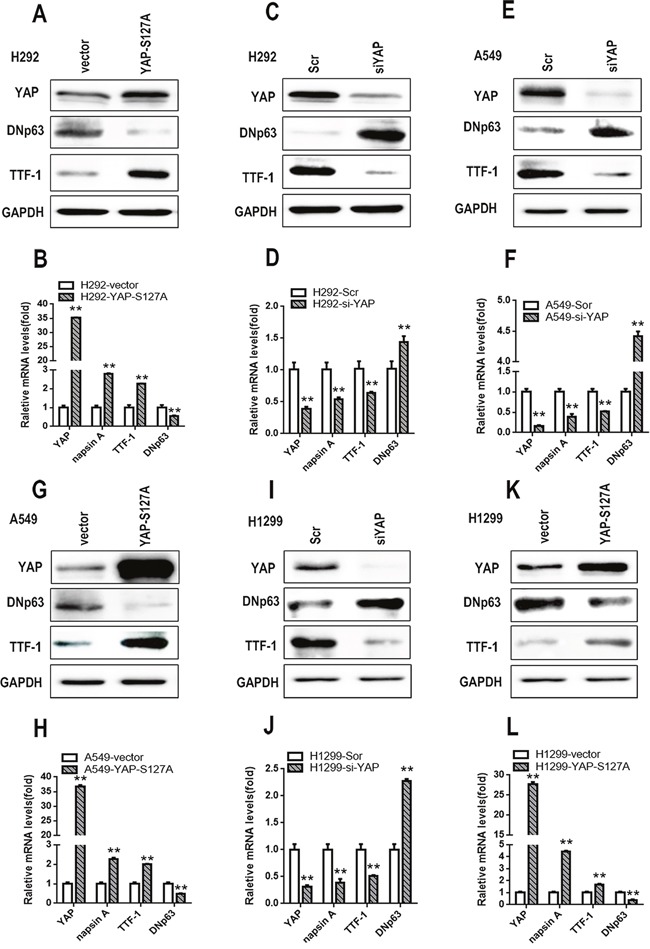
S100A7 and YAP have opposite effects on lung ADC to SCC transdifferentiation in lung cancer cells Following the ectopic expression YAP-S127A or knockdown of YAP in H292 cells, the expression of YAP, DNp63 and TTF-1 was examined by Western blotting **A, C**. and the expression of YAP, napsin A, TTF-1 and DNp63 was detected by real-time PCR **B, D**. Single silencing of YAP or overexpression of YAP-S127A in A549 **E, G**. or H1299 cells **I, K**. the expression of YAP, DNp63 and TTF-1 was detected by Western blotting. The expression of YAP, napsin A, TTF-1 and DNp63 was examined by real-time PCR **F, H, J, L**. **p < 0.01; t-test versus the YAP experimental groups and the control groups.

**Figure 7:**
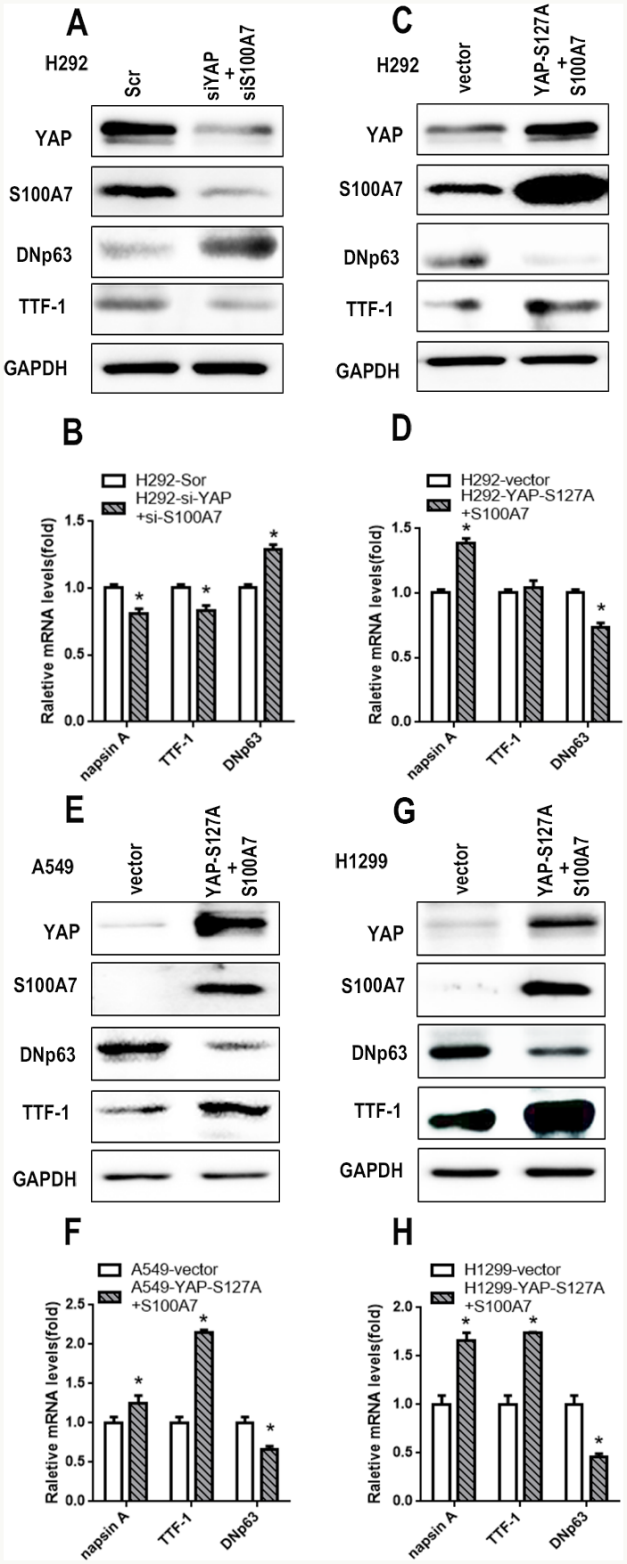
Ectopic expression of S100A7 partially rescues lung ADC to SCC transdifferentiation inhibited by YAP overexpression in lung cancer cells Following double depletion or the combined overexpression of S100A7 and YAP in H292 cells, the expression of S100A7, YAP, TTF-1 and DNp63 was examined by western blotting **A, C**. and the expression of napsin A, TTF-1 and DNp63 was determined by real-time PCR **B, D**. The combined overexpression of YAP and S100A7 in A549 cells **E**. and H1299 cells **G**. The transfection efficiency of both was examined by Western blotting. The expression of napsin A, TTF-1 and DNp63 was determined by real-time PCR F, H. GAPDH was used to assess equal loading. GAPDH assessed equal loading. *p < 0.05; t-test versus the experimental groups and the control groups.

Considering that S100A7 was inhibited by YAP and that the opposite effects of YAP and S100A7 were observed on lung ADC to SCC transition, we next explored the role of YAP in mediating S100A7 function during ADC to SCC transdifferentiation. We first performed experiments involving single depletion or overexpression of YAP in H292 cells. As expected, overexpression of YAP led to severe downregulation of S100A7 and DNp63 but significant upregulation of TTF1 and napsin A, whereas depletion of YAP alone inversely inhibited this transition in H292 cells (Figure 6A, 6B, 6C and 6D). To test whether this effect of YAP was cell-line specific, we also performed the same treatment in A549 and H1299 cells. Unsurprisingly, similar results were obtained in A549 cells (Figure 6E, 6F, 6G and 6H) and H1299 cells (Figure 6I, 6J, 6K and 6L), except with regard to S100A7. Strikingly, these results were consistent with reports that YAP overexpression or knockdown regulated lung ADC to SCC transdifferentiation in Lkb1-deficient mice [7]. Next, we performed a double depletion of S100A7 and YAP in H292 cells (Figure 7A). Interestingly, the combined abrogation of YAP and S100A7 showed the same tendency as the single removal of YAP but displayed weaker effects than single removal of YAP in H292 cells (Figure 7B), implying that YAP had a stronger influence on lung ADC to SCC transdifferentiation than S100A7. Strikingly, S100A7 overexpression partially overcame the inhibition of squamous transdifferentiation imposed by YAP overexpression in H292 cells (Figure 7C and 7D). Importantly, similar results were also obtained when both S100A7 and YAP were overexpressed in A549 (Figure 7E and 7F) and H1299 cells (Figure 7G and 7H). These data collectively provide evidence that S100A7 plays an important role in the initiation of squamous reprogramming downstream of YAP.

## DISCUSSION

Phenotypic transition or phenotypic plasticity is an important feature of tumors arising in various organs. Notably, multiple lines of evidence from previous studies have demonstrated that lung cancer cells are capable of carrying out lineage transition of cancer types or subtypes and thus exhibit phenotypic plasticity [1, 2, 3]. It has been reported that Nkx2-1 deletion in neoplastic lung tissue causes conversion to a gastric lineage [36]. The transition from a neuroendocrine to a mesenchymal phenotype can also be achieved by ectopic expression of oncogenic Ras ^V12^ in SCLC [37]. A recent study revealed the genotypic and histological transition of EGFR-mutant NSCLC into SCLC after targeted therapy [38]. Lung ADC with Lkb1 deficiency can progressively transdifferentiate into SCC, and depletion of YAP significantly accelerates this transition [6, 7]. Here, we demonstrated that S100A7 not only promotes lung ADC to SCC transdifferentiation in several lung cancer cells but also that its expression is differentially regulated by the Hippo-YAP pathway. Lung ADC and SCC are two distinct subtypes of lung cancer with different morphologies and different gene expression signatures. The transcription factor Nkx-2/TTF1 has emerged as a candidate regulator of lung adenocarcinoma differentiation and is expressed in 75-85% of human lung adenocarcinomas [6, 7, 39, 40]. Napsin A is an aspartic proteinase expressed in the lung and kidney. Among the three proteins currently used as markers for lung adenocarcinoma, TFF1 showed the same sensitivity (84.6%) as napsin A for adenocarcinoma, whereas surfactant protein-A and surfactant protein-B showed lower sensitivities. Moreover, napsin A showed the highest specificity (94.3%) for adenocarcinoma in non-small cell lung carcinoma [39, 40]. However, DNp63 is a marker of basal cells and is upregulated in SCC [40, 41]. We found that depletion of S100A7 markedly suppressed SCC marker (DNp63) expression but significantly promoted the expression of two markers for lung ADC (TTF1 and napsin A) in H292 cells. Importantly, the opposite results were obtained by S100A7 overexpression in H292, A549 and H1299 cells. Although the latent differentiation program of cancer cells is commonly repressed *in vitro*, our results provide direct evidence that lung ADC to SCC transdifferentiation is successfully rescued by S100A7 overexpression in several lung cancer cell lines.

YAP not only plays an important role in controlling lung cancer plasticity but also acts as a negative regulator in lung ADC to SCC transdifferentiation through DNp63 repression [7]. Considering the opposite effects of YAP and S100A7 on lung ADC to SCC transition, we further investigated the relationship of these two proteins in lung cancer cells. Importantly, we found that activation of the Hippo pathway promoted S100A7 expression in H292 cells, whereas inactivation of the Hippo pathway inhibited S100A7. Strikingly, S100A7 induction was always inversely correlated with YAP activity in the above-mentioned conditions. However, S100A7 was not successfully induced by YAP knockdown in A549 and H1299 cells. These data suggest that activation of the Hippo pathway is a necessary condition, but not an essential one, to induce S100A7 in lung cancer cells. We speculated that one possible factor was whether endogenous S100A7 was expressed in a cell line, which could primarily depend on the final differentiation state acquired by the cancer cell line. Several lines of evidence supported our hypothesis. First, lung H226 and H520 cells not only lacked S100A7 expression but also could not be induced to express S100A7 through YAP depletion. Moreover, we found that H226 cells formed poorly differentiated tumors in BALB/c nude mice (our unpublished data). Second, both S100A7 expression and induction occurred in HCC94 cells that displayed well-differentiated tumors in BALB/c nude mice [21]. Third, breast adenocarcinoma MDA-MB-468 cells highly expressed S100A7, whereas MDA-MB-231 cells did not express S100A7 and formed poorly differentiated tumors in ALS-treated BALB/c mice [42]. Fourth, S100A7 expression was positively correlated with the degree of differentiation in almost all types of SCC tissues, including lung SCC specimens [18, 19, 21]. Of course, additional genetic or epigenetic changes may also be required for S100A7 induction, but the precise mechanism is beyond the scope of this paper. Although we are not entirely clear on how S100A7 regulates DNp63, TFF1 and napsin A expression in lung cancer cells, our findings provide a novel pathway of lung cancer phenotypic plasticity during tumor progression.

In conclusion, our results demonstrate that S100A7 not only facilitates ADC to SCC transdifferentiation in lung cancer cells but also elicits a distinct response to Hippo-YAP regulation. Moreover, we speculate that active differentiation programs may influence lung cancer therapeutics. Our data also imply that the microenvironment of cancer cells might influence cellular plasticity and subtype transdifferentiation during metastasis.

## MATERIALS AND METHODS

### Cell culture

The human adenocarcinoma carcinoma cell lines H292, A549 and H1299 were purchased from the Chinese Academy of Sciences Committee Type Culture Collection Cell Bank and were authenticated by short tandem repeat analysis at HK Gene Science Technology Co. (Beijing, China). All cells were cultured according to the corresponding culture methods of the ATCC. Cell suspension cultures were obtained as described in our previous studies [11]. H292 cells were transfected with the indicated siRNAs simultaneously and then were cultured under suspension conditions for two days before the cells were harvested for western blotting.

### Western blotting

Western blotting analysis was performed as previously described [19]. The following antibodies were used: S100A7 (1/1000; Abcam, ab13680); YAP (1/500, Santa Cruz, sc-101199); pYAP (S127) (1/1000; Cell Signaling Technology, 13008S); anti-Flag tag (CWBIO, CW0287A); and anti-His tag (MBL, D291-3). GAPDH (ZSGB-BIO, TA-08) was used as a loading control.

### Reverse transcription and real-time PCR

Real-time PCR was performed on an ABI 7300 Real-time PCR System (Applied Biosystems) using Power SYBR Green PCR Master Mix (Applied Biosystems, Forster City, CA, USA) in a final volume of 20 μl. GAPDH was used as an endogenous control for each sample. The primers used for each of the genes are listed [Supplementary Table 1 and Supplementary Table 2].

### Plasmids and reagents

The pcDNA4-His-YAP WT and S127A vectors and the pCMV14-Flag-YAP WT and S94A vectors were kindly provided by Dr. Zhang (Mayo Clinic College of Medicine, USA). For pCMV14-Flag-LATS1, the LATS1 (NCBI Gene ID: 9113) cDNA fragment was amplified using 5′-CGGGGTACCATGAAGAGGAGTGAAAAG-3′ and 5′-GCTCTAGAAACATATACTAGATCGCGATTT-3′ and was then cloned into the mammalian expression vector pCMV14 (Invitrogen, Carlsbad, CA, USA) using the KpnI and XbaI restriction enzymes (Takara). Latrunculin B (L5288) and cytochalasin D (C8273) were purchased from Sigma.

### Statistical analysis

Statistical analysis was performed using GraphPad Prism software. Statistical significance was evaluated using Student's t-test (2-tailed) to compare two groups of data. The asterisks indicate significant differences between the experimental groups and the corresponding control condition. Differences were considered statistically significant at P-values of less than 0.05. P-values <0.05 and <0.01 are indicated with one and two asterisks, respectively.

## SUPPLEMENTARY MATERIALS FIGURES AND TABLES


