# Supplementary material for: S100A7 promotes lung adenocarcinoma to squamous carcinoma transdifferentiation, and its expression is differentially regulated by the Hippo-YAP pathway in lung cancer cells
Source: Oncotarget. 2017 Feb 3;8(15):24804–14. doi: 10.18632/oncotarget.15063 (PMC5421890; doi:10.18632/oncotarget.15063)
Supplement: Supplementary file 1 [file oncotarget-08-24804-s001.pdf]

S100A7 promotes lung adenocarcinoma to squamous carcinoma transdifferentiation, and its expression is differentially regulated by the Hippo-YAP pathway in lung cancer cells

SUPPLEMENTARY TABLES

Supplementary Table 1: siRNA sequences

Genes name	siRNA sequences
YAP-sense	5’GGUGAUACUAUCAACCAAATT 3’
YAP-antisense	5’UUUGGUUGAUAGUAUCACCTT 3’
LATS1-sense	5’GAGCUGGAAAGGUUCUAAATT 3’
LATS1-antisense	5’UUUAGAACCUUUCCAGCUCTT 3’
MST1-sense	5’GGACCUGCAUCAUGAACAATT 3’
MST1-antisense	5’UUGUUCAUGAUGCAGGUCCTT 3’
TEAD1-sense	5’GCCACUGCCAUUCAUAACATT 3’
TEAD1-antisense	5’UGUUAUGAAUGGCAGUGGCTT 3’
TEAD2-sense	5’GCCAGAUGCAGUUGAUUCUTT 3’
TEAD2-antisense	5’AGAAUCAACUGCAUCUGGCTT 3’
TEAD3-sense	5’CCAGUGUCCUGAAGAACAATT 3’
TEAD3-antisense	5’UUGUUCUGCAGGACACUGGTT 3’
TEAD4-sense	5’CCACGAAGGUCUGCUCUUUTT 3’
TEAD4-antisense	5’AAAGAGCAGACCUUCGUGGTT 3’
GSN-sense	5' CUGGGUUGGAAAGGAUUCUTT 3
GSN-antisens	5' AGAAUCCUUUCCAACCCAGTT 3'
CFL1-sense	5' CCACCUUUGUCAAGAUGCUTT 3'
CFL1-antisense	5' AGCAUCUUGACAAAGGUGGTT 3'
CAPZB-sense	5' GCUGGAGUGAUCCUCAUAATT 3'
CAPZB-antisense	5' UUAUGAGGAUCACUCCAGCTT 3'

Supplementary Table 2: Primers used for qPCR

Genes name	Primers sequences
S100A7-sense	5’CTTCCCCAACTTCCTTAGTG 3’
S100A7-antisense	5’GTAGTCTGTGGCTATGTCTC 3’
CYR61-sense	5’GCTGCGAGGAGTGGGTCTGT 3’
CYR61-antisense	5’GGGTTGTATAGGATGCGAGGCT 3’
CTGF-sense	5’GCATCCGTACTCCCAAAATCTC 3’
CTGF-antisense	5’CAGGGCACTTGAACTCCACC 3’
GAPDH-sense	5’GAGTCAACGGATTTGGTCGT 3’
GAPDH-antisense	5’GACAAGCTTCCCGTTCTCAG 3’
YAP-sense	5’CCTCTATTTGCTCTTCCTTGTCC 3’
YAP-antisense	5’CCATCATCCAAACAGGCTCAC 3’
LATS1-sense	5’CACCCTTCTTGGATACCACAGC 3’
LATS1-antisense	5’CTGATTGACTCGTATGGAGGAACA 3’
MST1-sense	5’TGCTTCTGACTCAATGCTTAGGG 3’
MST1-antisense	5’TGGCTGCTCACGTTGTAGTGG 3’
TEAD1-sense	5’TCGAGCAGCAGCGAGACCCAGACTC3’
TEAD1-antisense	5’TTACGAGGAAGAAGGCATTTTGAGG3’
TEAD2-sense	5’ TGCCTTCTTCCTGGTCAAGTTCTG 3’
TEAD2-antisense	5’ CTCATACTGGCTGCTCACTCCGT 3’
TEAD3-sense	5’ TCCTGTCAGACGAGGGCAAGATG 3’
TEAD3-antisense	5’ CTTCCGAGCTAGAACCTGTATGTG 3’
TEAD4-sense	5’ TTGAGCAGAGTTTCCAGGAGGCC 3’
TEAD4-antisense	5’ CAATCAGCTCGTTCCGACCATACA 3’
Napsin A-sense	5’ ATGGAACTGGGCGGGTAGAT 3’
Napsin A-antisense	5’ CAGAATGGGAAAACCGAGGC 3’
TTF-sense	5’ TCGCTGAGGTCAAGGCCCAATA 3’
TTF-antisense	5’ ACTGCTGCTGAGCCTGTTGC 3’
DNp63-sense	5’ CAGACTCAATTTAGTGAG 3’
DNp63-antisense	5’ AGCTCATGGTTGGGGCA 3’
CFL-1-sense	5'-ACCCCTACGCCACCTTTGTC-3'
CFL-1-antisense	5'-TCATGCTTGATCCCTGTCAGCT-3'
CAPZB-sense	5'-TAATCCTTTCCCACCACCTTGT-3'
CAPZB -antisense	5'-CTGGACTGTGCCTTGGACCTAA-3'
GSN-sense	5’TCCAACGATGCCTTTGTTCTGA3’
GSN-antisense	5’CATCTGGCTCGCTGCCTTCT3’
